# Long-chain dicarboxylic acids play a critical role in inducing peroxisomal β-oxidation and hepatic triacylglycerol accumulation

**DOI:** 10.1016/j.jbc.2023.105174

**Published:** 2023-08-19

**Authors:** Wei Zhang, Lina Zhang, Haoya Yao, Yaoqing Wang, Xiao Zhang, Lin Shang, Xiaocui Chen, Jia Zeng

**Affiliations:** School of Life Science, Hunan University of Science and Technology, Xiangtan, Hunan, P. R. China

**Keywords:** dicarboxylic acids, ω-oxidation, peroxisomes, mitochondria, hepatic steatosis

## Abstract

Recent studies provide evidence that peroxisomal β-oxidation negatively regulates mitochondrial fatty acid oxidation, and induction of peroxisomal β-oxidation causes hepatic lipid accumulation. However, whether there exists a triggering mechanism inducing peroxisomal β-oxidation is not clear. Long-chain dicarboxylic acids (LCDAs) are the product of mono fatty acids subjected to ω-oxidation, and both fatty acid ω-oxidation and peroxisomal β-oxidation are induced under ketogenic conditions, indicating there might be a crosstalk between. Here, we revealed that administration of LCDAs strongly induces peroxisomal fatty acid β-oxidation and causes hepatic steatosis in mice through the metabolites acetyl-CoA and hydrogen peroxide. Under ketogenic conditions, upregulation of fatty acid ω-oxidation resulted in increased generation of LCDAs and induction of peroxisomal β-oxidation, which causes hepatic accumulation of lipid droplets in animals. Inhibition of fatty acid ω-oxidation reduced LCDA formation and significantly lowered peroxisomal β-oxidation and improved hepatic steatosis. Our results suggest that endogenous LCDAs act as triggering molecules inducing peroxisomal β-oxidation and hepatic triacylglycerol deposition. Targeting fatty acid ω-oxidation might be an effective pathway in treating fatty liver and related metabolic diseases through regulating peroxisomal β-oxidation.

Peroxisomal fatty acid β-oxidation system was discovered in the 1970s and plays an important role in metabolism of long-chain and very long-chain fatty acids (VLCFAs) (1, 2, 3). For a long time, the pathophysiological roles of peroxisomal β-oxidation are not clear; until recently, multiple reports suggest that induction of peroxisomal β-oxidation plays a pathogenic role in inducing hepatic steatosis and related metabolic disorder through the metabolites acetyl-CoA and hydrogen peroxide (4, 5, 6, 7, 8, 9). Peroxisomal β-oxidation can be induced under the conditions of fasting, diabetes, obesity, high-fat diet (HFD) feeding, or hypolipidemic drugs (1, 10, 11, 12, 13, 14), which suggest that peroxisomal β-oxidation might be mediated through alterations in pathophysiological state and upregulated under ketogenic conditions, a metabolic state that ketone bodies (KBs) produced from fatty acid oxidation (FAO) serve as the main energy source. However, the potential mechanism by which increased supply of fatty acids and accelerated ketogenesis cause induction of peroxisomal β-oxidation is not clear, whether there exists a triggering mechanism inducing peroxisomal fatty acid β-oxidation remains to be resolved.

To explore the potential mechanism, we shed light on long-chain dicarboxylic acids (LC-DCAs), the product of mono fatty acids subjected to ω-oxidation (15). In mammalians, LC-DCAs are generated from mono fatty acids that are catalyzed first by CYP4A1, followed by alcohol dehydrogenase (ADH) and aldehyde dehydrogenase (15, 16, 17). Although the crosstalk between fatty acid ω-oxidation and peroxisomal β-oxidation is not established so far, we noted the fact that clofibrate (CFB), a well-known drug that strongly stimulates peroxisomal β-oxidation, also remarkably induces fatty acid ω-oxidation (18, 19, 20). Most importantly, it was reported that upregulation in fatty acid ω-oxidation resulted in induction of peroxisomal β-oxidation, whereas suppression of fatty acid ω-oxidation significantly lowered peroxisomal FAO in isolated hepatocytes treated with CFB (20). Therefore, we hypothesized that LC-DCAs might play a role in inducing peroxisomal β-oxidation and further leads to hepatic lipid accumulation in animals.

This study investigated the effects of LC-DCAs on peroxisomal FAO and explored the potential mechanism by which endogenous LC-DCAs induced peroxisomal β-oxidation and hepatic steatosis in animals under ketogenic conditions.

## Results

### Administration of LC-DCAs strongly induces peroxisomal β-oxidation in mouse liver

**Figure 1:**
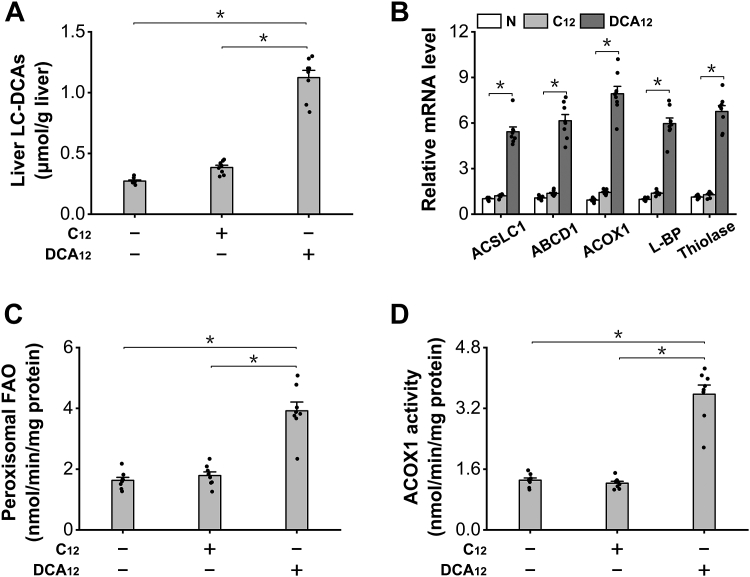
**Administration of DCA**_**12**_**strongly induces peroxisomal β-oxidation in C57BL/6 mice.***A*, liver LC-DCAs increased significantly in the mice treated with DCA_12_. *B*, mRNA expressions of the enzymes involved in peroxisomal β-oxidation were upregulated in the livers of the mice treated with DCA_12_, whereas no changes were observed in C12 group. *C*, peroxisomal β-oxidation was remarkably enhanced in the livers of DCA_12_-treated mice, whereas no changes were observed in C12 group. *D*, the activity of ACOX1 increased significantly in the livers of DCA_12_-treated mice. ∗*p* < 0.05 by *t* test between paired groups. n = 8. ACOX1, acyl-CoA oxidase-1; C12, lauric acid; DCA_12_, dodecanedioic acid; LC-DCA, long-chain dicarboxylic acid.

LC-DCAs are dicarboxylic acids with carbon chain length ≧10, as a typical and widely used LC-DCA, DCA_12_ (dodecanedioic acid), was administered to C57BL/6 mice to determine whether this kind of fatty acid was able to induce peroxisomal FAO *in vivo*, and the mice fed lauric acid (C12) were used as a negative control. We measured hepatic LC-DCAs and observed significant elevation in the livers of the mice fed DCA_12_ (Fig. 1*A*). Administration of DCA_12_ to the mice for 7 days strongly induced mRNA expression levels of the enzymes in peroxisomal β-oxidation, whereas no alterations were shown in C12 group, as shown in Figure 1*B*. The activity of peroxisomal β-oxidation, and acyl-CoA oxidase-1 (ACOX1), the rate-limiting enzyme in peroxisomal β-oxidation, was measured, and the results indicated that the activities of both peroxisomal β-oxidation and ACOX1 were remarkably enhanced in the livers of DCA_12_-treated mice, whereas no changes in C12 group occurred (Fig. 1, *C* and *D*). Therefore, the results indicated that DCA_12_ treatment robustly enhanced peroxisomal fatty acid β-oxidation in mouse liver.

### LC-DCA treatment increased acetyl-CoA generation *via* induction of peroxisomal β-oxidation

**Figure 2:**
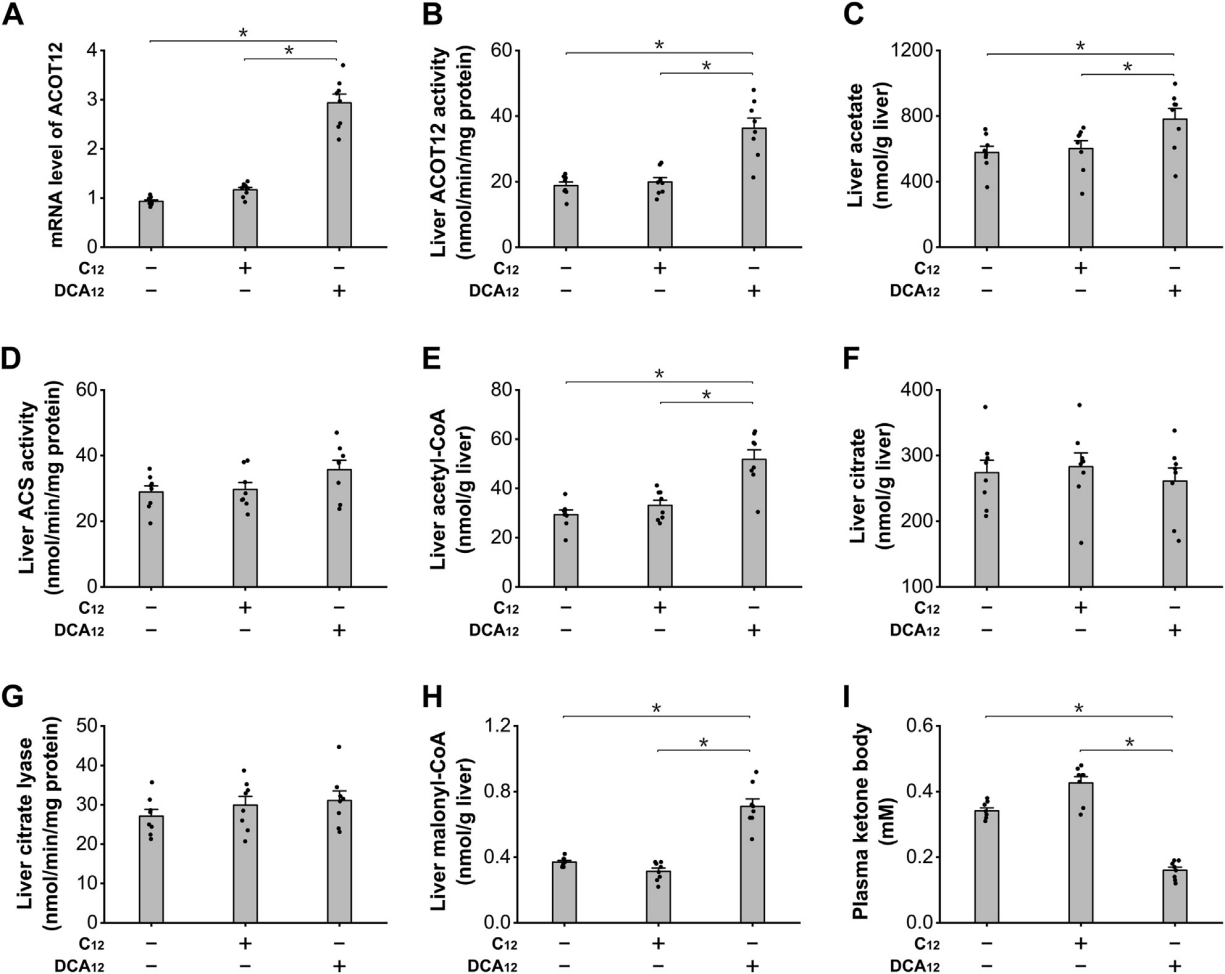
**DCA**_**12**_**treatment caused suppression of mitochondrial FAO through increasing malonyl-CoA formation.***A*, administration of DCA_12_ significantly increased mRNA expression of liver ACOT12 in mice. *B*, liver ACOT12 activity increased significantly in the DCA_12_-fed mice. *C*, liver acetate increased significantly in the mice treated with DCA_1,_ whereas no alteration was observed in the C12 group. *D*, liver ACS activity was not changed among all the groups. *E*, liver acetyl-CoA increased significantly in the liver of the mice treated with DCA_12_. *F*, liver citrate was not altered among all the groups. *G*, liver citrate lyase activity was not changed significantly among all the groups. *H*, administration of DCA_12_ significantly increased liver malonyl-CoA content, whereas no alteration in the C12 group was observed. *I*, plasma KB was significantly lower in the mice treated with DCA_12_. ∗*p* < 0.05 by *t* test between paired groups. n = 8. ACS, acetyl-CoA synthetase; DCA_12_, dodecanedioic acid; FAO, fatty acid oxidation; KB, ketone body.

Previous studies indicated that peroxisomal β-oxidation of fatty acids generated considerable free acetate as the product (21, 22, 23), and the released acetate can be used for synthesis of acetyl-CoA and malonyl-CoA, thereby negatively regulating mitochondrial FAO (24, 25). Therefore, DCA_12_ treatment might affect mitochondrial FAO through the metabolite acetyl-CoA. ACOT12 is a specific acetyl-CoA thioesterase in peroxisomes; chronic administration of DCA_12_ significantly increased liver ACOT12 expression and activity, as shown in Figure 2, *A* and *B*. Increased acetyl-CoA formation and accelerated hydrolysis of acetyl-CoA led to significant increase in acetate content in the liver of the mice treated with DCA_12_ (Fig. 2*C*). Liver acetyl-CoA synthetase (ACS) increased slightly in the DCA_12_-fed mice (Fig. 2*D*), which resulted in increased formation of acetyl-CoA in mouse liver, as shown in Figure 2*E*. The increased formation of acetyl-CoA after treatment of DCA_12_ was not attributed to alterations of liver citrate or citrate lyase as both liver citrate or citrate lyase activity was not changed significantly among all the groups, as shown in Figure 2, *F* and *G*. Liver malonyl-CoA was measured, and the results indicate that administration of DCA_12_ significantly increased liver malonyl-CoA level (Fig. 2*H*). It is well known that mitochondrial FAO was controlled by intracellular malonyl-CoA, and elevation in malonyl-CoA level causes suppression of mitochondrial FAO and lipid accumulation (26). DCA_12_ treatment for 7 days significantly lowered plasma KB, a measure of mitochondrial FAO, as shown in Figure 2*I*. The results suggested that DCA_12_ treatment significantly increased liver acetyl-CoA and malonyl-CoA content and caused suppression of mitochondrial FAO through induction of peroxisomal β-oxidation.

### LC-DCA treatment increased hydrogen peroxide formation and suppressed adipose triglyceride lipase activity

**Figure 3:**
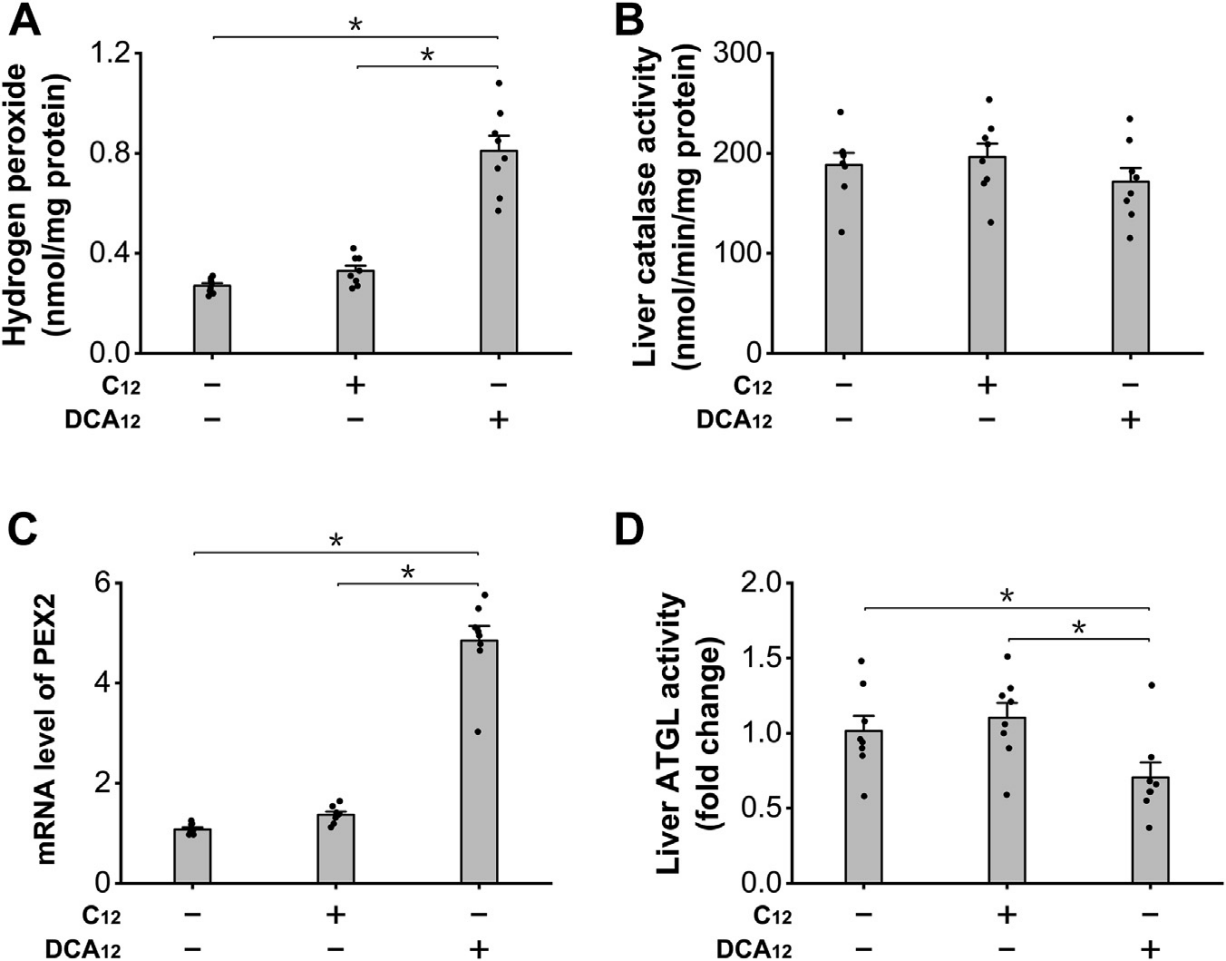
**DCA**_**12**_**treatment increased hydrogen peroxide formation and suppressed liver ATGL activity.***A*, DCA_12_ treatment significantly increased liver hydrogen peroxide level, whereas no alteration was observed in C12 group. *B*, liver catalase was not altered among all the groups. *C*, PEX2 expression was upregulated in the liver of the mice treated with DCA_12_. *D*, liver ATGL activity decreased significantly in DCA_12_-treated mice, whereas no change was observed in the C12 group. ∗*p* < 0.05 by *t* test between paired groups. n = 8. ATGL, adipose triglyceride lipase; DCA_12_, dodecanedioic acid; PEX2, peroxin 2.

Hydrogen peroxide is a byproduct in peroxisomal β-oxidation, and recently, hydrogen peroxide derived from peroxisomal β-oxidation was identified to play a role in the development of hepatic lipid accumulation through mediating liver peroxin 2 (PEX2) expression and adipose triglyceride lipase (ATGL) activity (9). As DCA_12_ treatment significantly enhanced peroxisomal FAO, it was expected that liver ATGL activity might be affected by DCA through the aforementioned mechanism. Liver hydrogen peroxide level was determined, and the results indicated that administration of DCA_12_ significantly increased liver hydrogen peroxide level, whereas no significant increase was observed in the mice treated with C12, as shown in Figure 3*A*. The increase in hydrogen peroxide was attributed to the enhanced peroxisomal β-oxidation rather than suppression of catalase as the activity of liver catalase was not affected by DCA_12_ (Fig. 3*B*). Liver PEX2 expression was upregulated in the mice treated with DCA_12,_ and liver ATGL decreased in the DCA_12_-treated mice, whereas no alterations were shown in the C12 group, as shown in Figure 3, *C* and *D*. The results provided evidence that induction of peroxisomal β-oxidation by DCA_12_ resulted in excessive generation of hydrogen peroxide and caused suppression of liver ATGL, which might lead to liver accumulation of lipid droplets.

### Administration of LC-DCAs causes hepatic accumulation of lipid droplets

**Figure 4:**
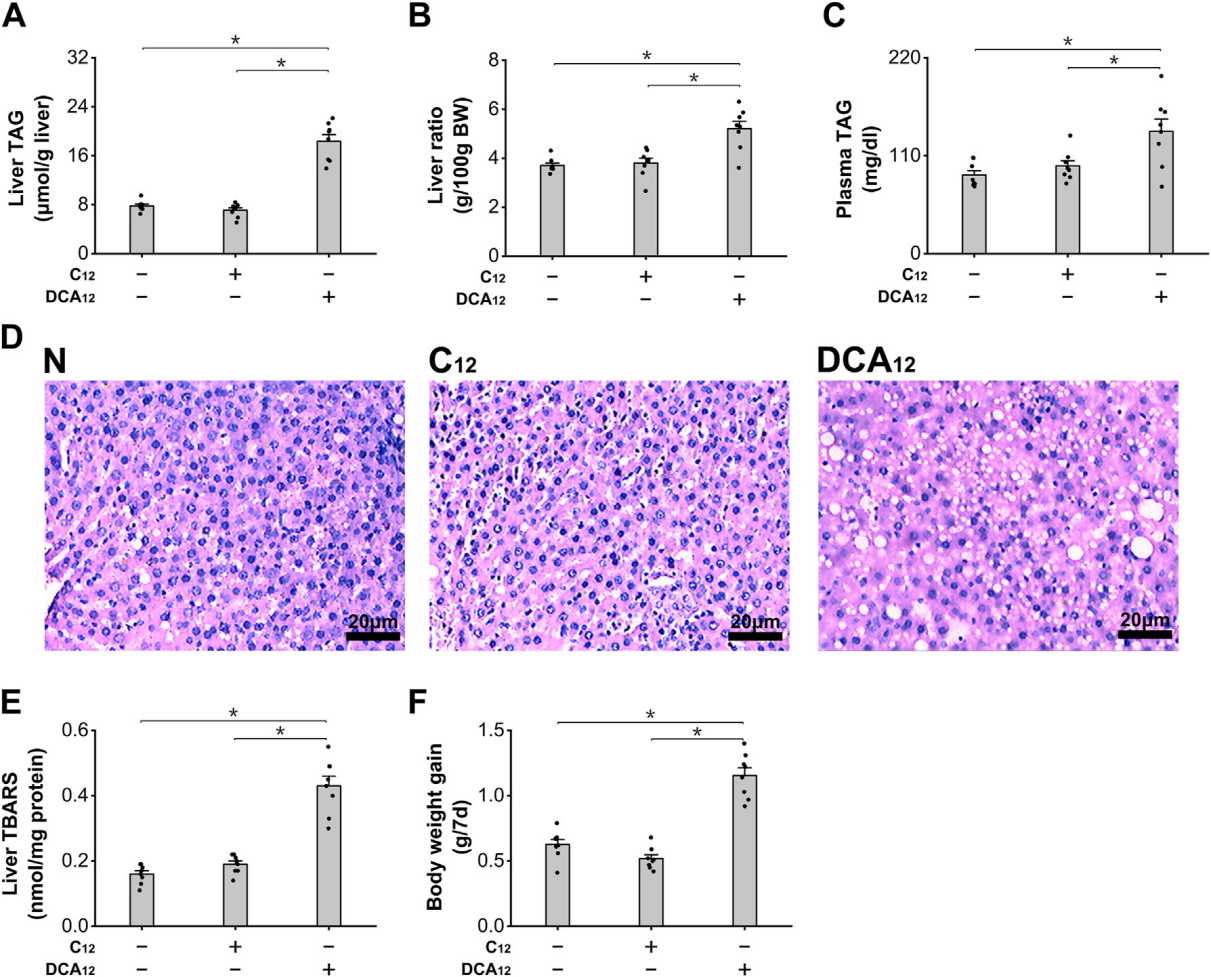
**Administration of DCA**_**12**_**induced hepatic accumulation of lipid droplets.***A*, administration of DCA_12_ significantly increased liver TAG content in the mice, whereas no alteration was observed in the C12 group. *B*, liver ratio was significantly higher in the mice treated with DCA_12_. *C*, plasma TAG was significantly increased in the mice treated with DCA_12_. *D*, lipid droplets increased significantly in liver sections of the mice treated with DCA_12_, whereas no change was observed in the C12 group. *E*, liver TBARS increased significantly in the mice treated with DCA_12_. *F*, body weight gain was significantly higher in the mice treated with DCA_12_. ∗*p* < 0.05 by *t* test between paired groups. n = 8. C12, lauric acid; DCA_12_, dodecanedioic acid; TAG, triacylglycerol; TBARS, thiobarbituric acid–reactive substance.

Administration of DCA_12_ significantly increased liver triacylglycerol (TAG) in C57BL/6 mice, whereas C12 showed no significant effect on liver TAG compared with the normal mice (Fig. 4*A*). Liver to body weight ratio increased significantly in the mice treated with DCA_12_ because of lipid droplet accumulation and possibly proliferation of peroxisomes, whereas no alteration was seen in the mice after treatment with C12 (Fig. 4*B*). Plasma TAG was significantly higher in the mice treated with DCA_12_ (Fig. 4*C*). Liver sections with hematoxylin–eosin staining indicated that significant increases in lipid droplets were observed in the liver of the mice treated with DCA_12_, whereas no alteration was observed in the C12-treated group, as shown in Figure 4*D*. Thiobarbituric acid–reactive substance as a marker for oxidative stress increased significantly in the liver of the DCA_12_-fed mice, whereas no alterations were observed in the mice treated with C12, as shown in Figure 4*E*. Body weight gain was significantly higher in the mice treated with DCA_12_ compared with control group, as shown in Figure 4*F*. The results suggested that administration of LC-DCAs caused hepatic TAG accumulation and oxidative stress through induction of peroxisomal β-oxidation.

### Fatty acid ω-oxidation was induced in mice under ketogenic conditions

**Figure 5:**
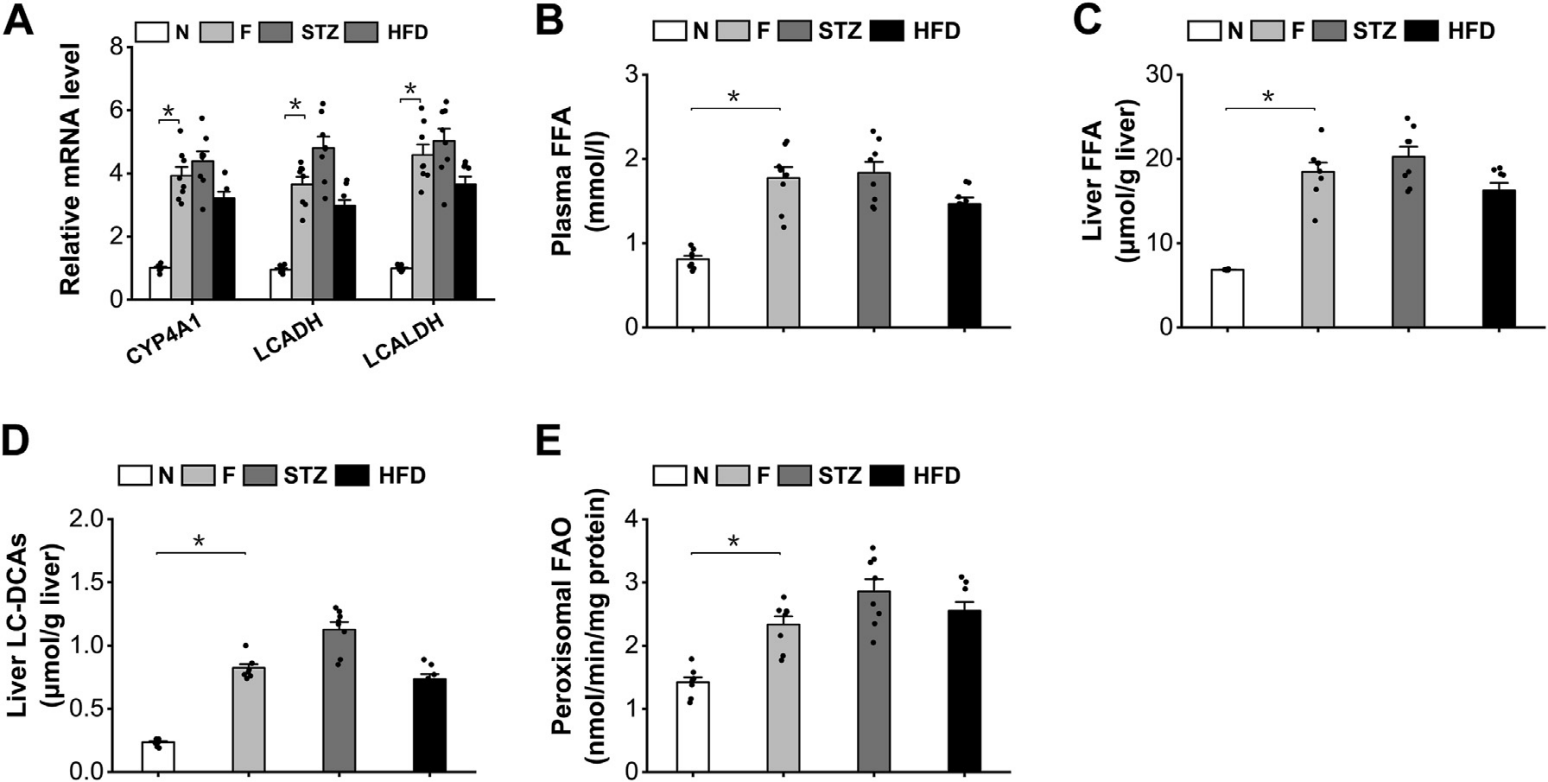
**Fatty acid ω-oxidation was induced in the mice under ketogenic conditions.***A*, mRNA expressions of the key enzymes in fatty acid ω-oxidation were upregulated in the liver of the fasting (F), diabetic, and HFD-fed mice. *B*, plasma FFA level was significantly elevated in the fasting, diabetic, and HFD-fed mice. *C*, liver FFA increased significantly in the fasting, diabetic, and HFD-fed mice compared with the normal mice. *D*, the content of LC-DCAs increased significantly in the liver of the fasting, diabetic, and HFD-fed mice. *E*, peroxisomal β-oxidation was significantly enhanced in the liver of the fasting, diabetic, and HFD-fed mice. ∗*p* < 0.05 by *t* test between paired groups. n = 8. FFA, free fatty acid; HFD, high-fat diet; LC-DCA, long-chain dicarboxylic acid; STZ, streptozocin.

To explore the potential pathophysiological roles of endogenous LC-DCAs in regulating peroxisomal fatty acid metabolism and hepatic lipid homeostasis, we noted the fact that peroxisomal β-oxidation is induced under the condition of prolonged fasting, diabetes, and HFD feeding (4, 10, 11, 12, 13, 14), and endogenously generated LC-DCAs through ω-oxidation of excessive fatty acids might be involved in induction of peroxisomal FAO under ketogenic conditions. To confirm this, mRNA expressions of the key enzymes involved in fatty acid ω-oxidation in the livers of the mice under ketogenic condition were measured, and the results indicated that the expressions of the enzymes in fatty acid ω-oxidation were upregulated in the liver of the fasting, diabetes, and HFD-fed mice compared with normal control, as shown in Figure 5*A*. The induction of fatty acid ω-oxidation was attributed to elevation in plasma-free fatty acids (FFAs) and increased hepatic uptake of FFA in liver under ketogenic conditions (Fig. 5, *B* and *C*). The level of liver LC-DCAs was measured, which increased significantly in the fasting, diabetic, and HFD-fed mice, as shown in Figure 5*D*. As expected, peroxisomal β-oxidation was upregulated in the livers of the mice under the condition of fasting, diabetes, and HFD feeding (Fig. 5*E*). Therefore, both fatty acid ω-oxidation and peroxisomal β-oxidation were induced under ketogenic conditions, and endogenous LC-DCAs might play a role in regulating peroxisomal β-oxidation and mediating hepatic lipid homeostasis.

### Endogenously generated LC-DCAs regulate peroxisomal β-oxidation in fasting mice

**Figure 6:**
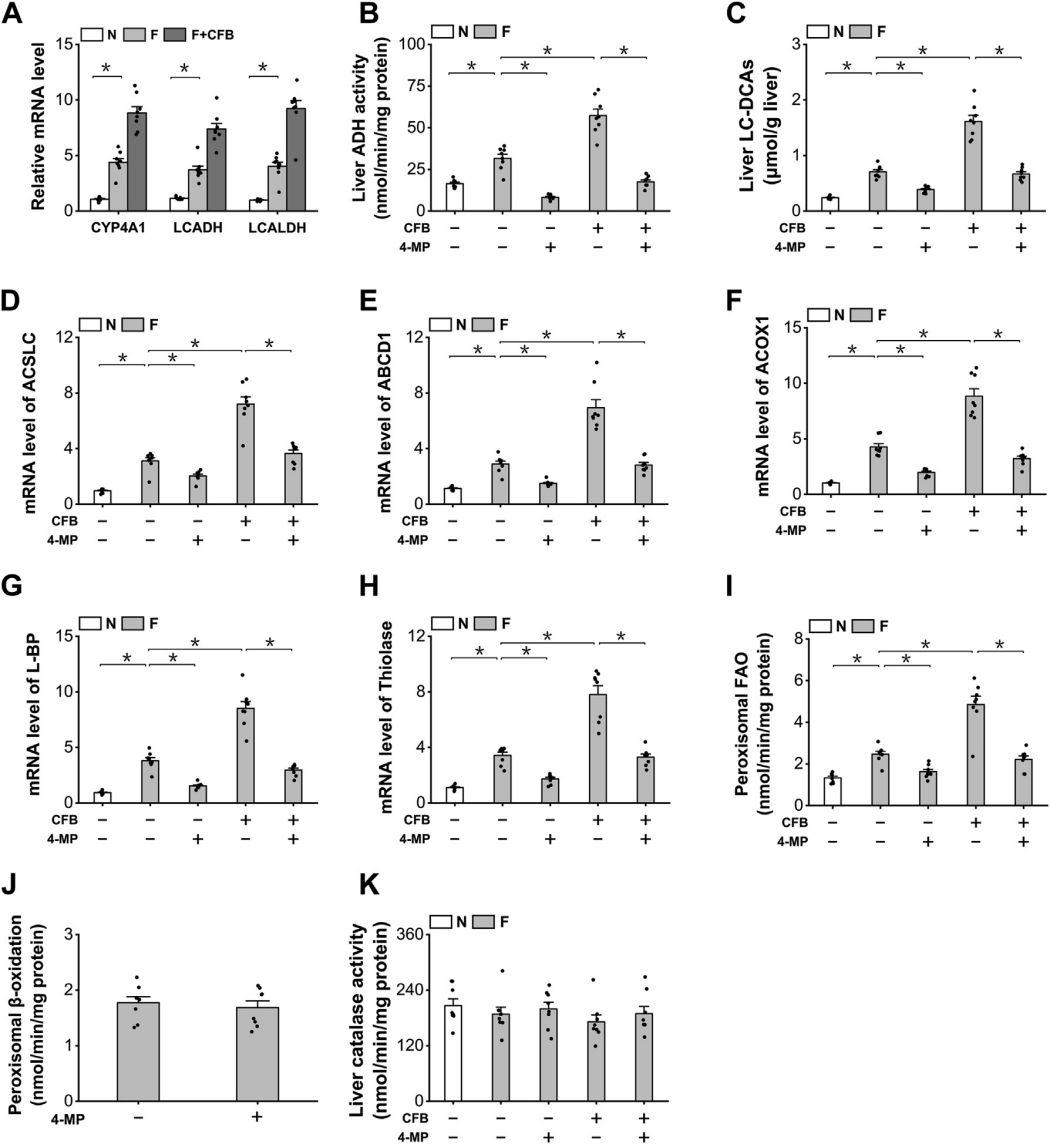
**Endogenously generated LC-DCAs regulated peroxisomal β-oxidation in fasting mice.***A*, CFB treatment strongly induced mRNA expression of the enzymes in fatty acid ω-oxidation in the liver of the fasting mice. *B*, administration of CFB remarkably increased liver ADH activity in the liver of the fasting mice, as suppressed by the treatment of 4-MP. *C*, CFB treatment significantly increased liver LC-DCA content in the liver of the fasting mice, whereas 4-MP treatment reduced DCA_12_ level in the fasting mice. *D*–*H*, mRNA expression of the key enzymes involved in peroxisomal β-oxidation was induced by CFB and downregulated by the treatment of 4-MP. *I*, CFB treatment remarkably enhanced liver peroxisomal β-oxidation in the fasting mice, as decreased by 4-MP. *J*, pretreatment with 4-MP did not inhibit peroxisome β-oxidation directly in isolated peroxisomes. *K*, liver catalase activity was not affected by the treatment of CFB or 4-MP. ∗*p* < 0.05 by *t* test between paired groups. n = 8. ADH, alcohol dehydrogenase; CFB, clofibrate; DCA_12_, dodecanedioic acid; LC-DCA, long-chain dicarboxylic acid; 4-MP, 4-methylpyrazole.

To determine whether alterations in endogenous LC-DCAs might regulate peroxisomal β-oxidation, we used CFB, a well-known drug to enhance fatty acid ω-oxidation and increase liver generation of LC-DCAs (18, 19, 20), and 4-methylpyrazole (4-MP), a specific inhibitor of ADH, to suppress fatty acid ω-oxidation and reduce generation of LC-DCAs in the fasting mice (27). mRNA expressions of the enzymes involved in fatty acid ω-oxidation were upregulated in the liver of the fasting mice compared with the fed animals, as further induced after treatment with CFB (Fig. 6*A*). The catalytic activity of long-chain ADH, a critical enzyme in generation of LC-DCAs, increased significantly in the liver of the fasting mice, as strongly enhanced by the treatment of CFB and abolished by the treatment of 4-MP, as shown in Figure 6*B*. LC-DCA content increased significantly in the liver of the fasting mice, as further increased in the liver of CFB-treated mice and significantly reduced after treatment with 4-MP, as shown in Figure 6*C*.

Administration of LC-DCAs induced peroxisomal β-oxidation in liver, as reflected by the mRNA expression levels of the enzymes involved in peroxisomal β-oxidation (Fig. 6, *D*–*H*). Peroxisomal β-oxidation was also determined, fasting significantly increased liver peroxisomal fatty acid β-oxidation, CFB treatment strongly enhanced peroxisomal β-oxidation activity, and administration of 4-MP significantly lowered peroxisomal β-oxidation in the fasting mice, as shown in Figure 6*I*. Isolated peroxisomes were incubated with 1 mM 4-MP, and then peroxisomal β-oxidation was assayed, and the results indicated that 4-MP did not cause direct suppression of peroxisomal β-oxidation, as shown in Figure 6*J*. Therefore, the decreased peroxisomal β-oxidation as caused by 4-MP was due to reduced generation of LC-DCAs in liver. Liver catalase activity was not affected by the treatment of CFB or 4-MP (Fig. 6*K*).

### Accumulation of endogenous LC-DCAs induces hepatic TAG accumulation in fasting mice

**Figure 7:**
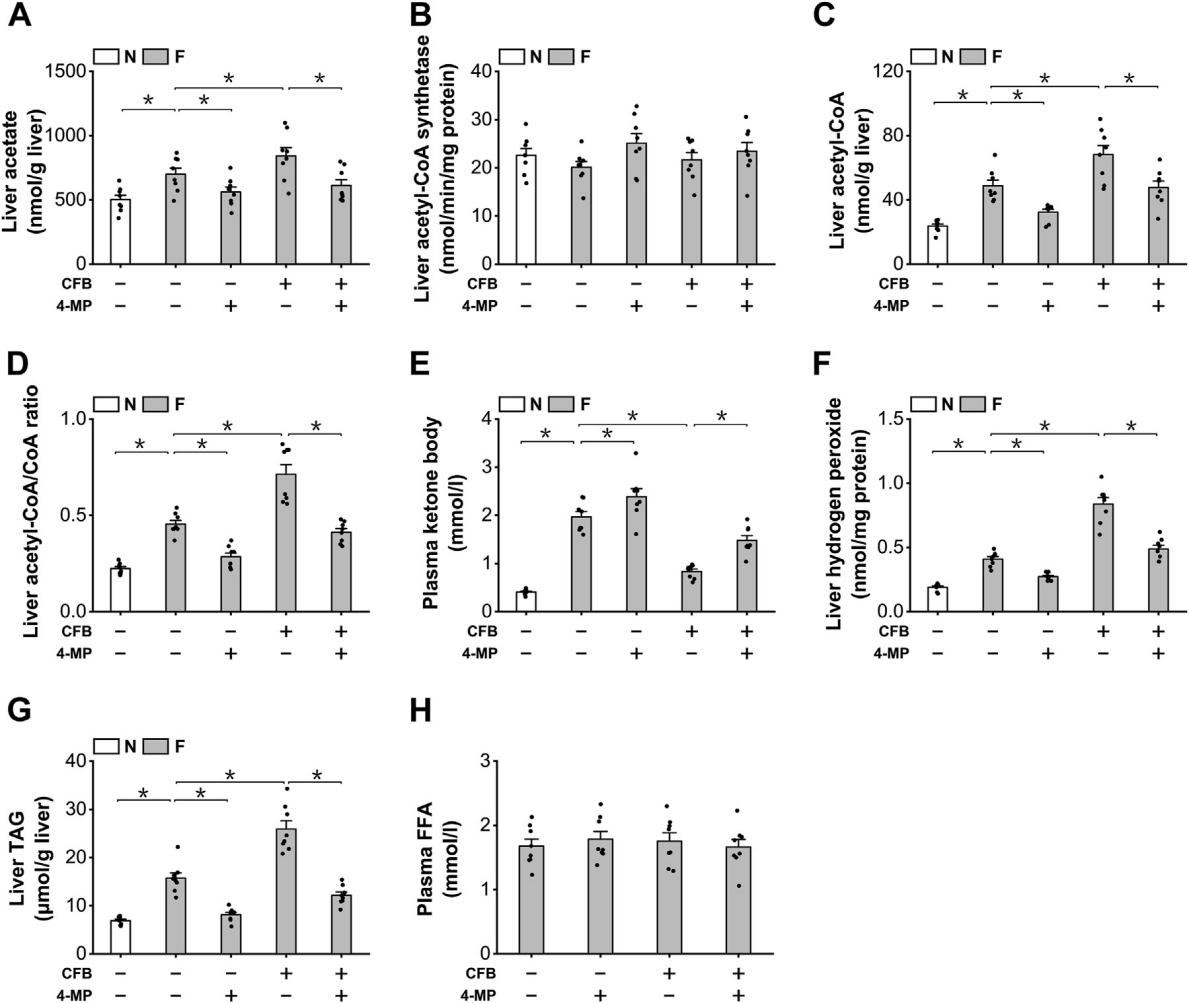
**Accumulation of endogenous LC-DCAs induced hepatic TAG accumulation in fasting mice.***A*, administration of CFB significantly increased liver acetate content in the fasting mice, as reduced by 4-MP. *B*, Liver ACS activity was not affected by the treatment of CFB or 4-MP. *C*, liver acetyl-CoA increased significantly in the fasting mice treated with CFB and reduced by 4-MP. *D*, liver acetyl-CoA/CoA ratio was significantly higher in the mice treated with CFB, which was lowered by the treatment of 4-MP. *E*, administration of CFB significantly decreased plasma ketone body in the fasting mice, as recovered by 4-MP. *F*, liver hydrogen peroxide was significantly higher in the fasting mice treated with CFB, as reduced by 4-MP. *G*, CFB treatment significantly increased liver TAG content in the fasting mice, which was reduced by the treatment of 4-MP. *H*, plasma free fatty acids was not changed by the treatment of CFB or 4-MP. ∗*p* < 0.05 by *t* test between paired groups. n = 8. 4-MP, 4-methylpyrazole; ACS, acetyl-CoA synthetase; CFB, clofibrate; LC-DCA, long-chain dicarboxylic acid; TAG, triacylglycerol.

As endogenously generated LC-DCAs induced peroxisomal β-oxidation, it was expected that endogenous LC-DCAs should play a role in regulating hepatic lipid homeostasis in the fasting mice through the metabolites derived from peroxisomal β-oxidation. Liver acetate was measured, and the content of free acetate was significantly higher in the livers of the fasting mice compared with the fed mice, as further increased by CFB and reduced by the treatment of 4-MP (Fig. 7*A*). Liver ACS activity was not affected by CFB or 4-MP (Fig. 7*B*). Liver acetyl-CoA level and acetyl-CoA/CoA ratio were then determined, and administration of CFB significantly increased liver acetyl-CoA content and acetyl-CoA/CoA ratio in the fasting mice, as decreased by the treatment of 4-MP (Fig. 7, *C* and *D*). Elevation of liver acetyl-CoA/CoA ratio has been reported to cause feedback inhibition of mitochondrial fatty acid β-oxidation (6, 28, 29). As a measure of mitochondrial FAO, plasma KB was determined, and the results indicated that administration of CFB significantly decreased plasma KB in the fasting mice, whereas 4-MP treatment significantly enhanced plasma KB level in the fasting and CFB-treated mice, as shown in Figure 7*E*. Therefore, endogenous LC-DCAs played a role in regulating mitochondrial FAO through the metabolites from peroxisomal β-oxidation. Hydrogen peroxide is a byproduct in peroxisomal β-oxidation, and CFB treatment significantly increased liver hydrogen peroxide in the fasting mice, as reduced by 4-MP (Fig. 7*F*.) Administration of CFB significantly increased liver TAG content in the fasting mice, which was reduced by 4-MP (Fig. 7*G*). Plasma FFA was not significantly changed among all the groups, as shown in Figure 7*H*. The results suggested that excessive generation of LC-DCAs caused hepatic TAG accumulation in the fasting mice through induction of peroxisomal β-oxidation, whereas suppression of fatty acid ω-oxidation improved hepatic steatosis by lowering peroxisomal fatty acid oxidation. Therefore, endogenous LC-DCAs played an important role in inducing peroxisomal β-oxidation and hepatic TAG accumulation under ketogenic conditions.

## Discussion

**Figure 8:**
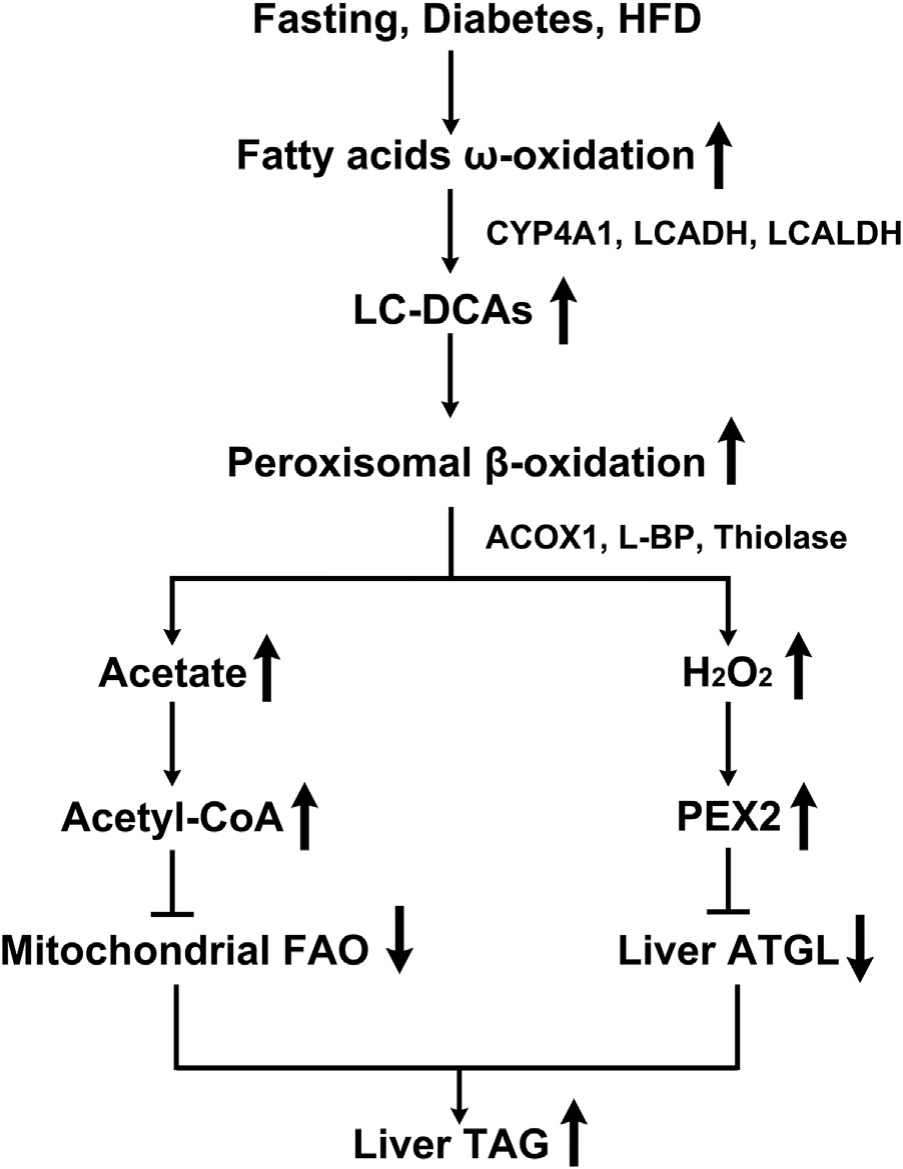
**Proposed mechanism by which endogenous LC-DCAs induce peroxisomal β-oxidation and hepatic TAG accumulation under ketogenic conditions**. Under ketogenic conditions, elevation in plasma FFA results in induction of fatty acid ω-oxidation, and hepatic LC-DCAs increase significantly, which act as stimulating molecules inducing peroxisomal β-oxidation. Excessive oxidation of fatty acids in peroxisomes releases considerable free acetate and hydrogen peroxide, which cause suppression of mitochondrial FAO and liver ATGL, and lead to hepatic TAG accumulation. ATGL, adipose triglyceride lipase; FAO, fatty acid oxidation; FFA, free fatty acid; LC-DCA, long-chain dicarboxylic acid; TAG, triacylglycerol.

This study demonstrates a role of LC-DCAs in inducing peroxisomal β-oxidation and a novel mechanism by which induction of fatty acid ω-oxidation and accumulation of endogenous LC-DCAs cause hepatic steatosis in animals under ketogenic conditions. The proposed mechanism is shown in Figure 8. Plasma FFAs are elevated significantly in animals under the conditions of fasting, diabetes, or HFD feeding, which results in increased hepatic uptake of fatty acids and the gene expressions involved in fatty acid ω-oxidation are induced and hepatic LC-DCAs increase significantly, which act as stimulating molecules inducing peroxisomal β-oxidation. Excessive oxidation of fatty acids in peroxisomes releases considerable free acetate and hydrogen peroxide, which cause suppression of mitochondrial FAO and liver ATGL, and lead to hepatic TAG accumulation. It is suggested that endogenous LC-DCAs play a pathogenic role in inducing hepatic steatosis and related metabolic disorder through upregulation of peroxisomal β-oxidation system.

Peroxisomal β-oxidation system plays an important role in metabolism of long-chain fatty acids and VLCFAs, and especially, VLCFA is metabolized exclusively in peroxisomes, and deficiency of peroxisomal FAO results in accumulation of VLCFA and causes Zellweger syndrome and X-linked adrenoleukodystrophy (3). On the other hand, multiple evidence suggest that excessive fatty acid oxidation in peroxisomes cause hepatic TAG accumulation and oxidative stress and might play an important role in the development of fatty liver and insulin resistance (IR) (4, 5, 6, 7, 8, 9). It was reported that the acetyl-CoA that generated in peroxisomal β-oxidation was used for synthesis of malonyl-CoA or causes feedback suppression of mitochondrial acyl-CoA dehydrogenase, thereby negatively regulating mitochondrial FAO (4, 5, 6). The acetyl-CoA derived from peroxisomal β-oxidation can also inhibit autophagy by promoting Raptor acetylation and mammalian target of rapamycin complex 1 activation, thereby causing hepatic lipid deposition (7, 8). Hydrogen peroxide, a byproduct in peroxisomal β-oxidation, was recently identified to play a role in regulating liver ATGL through mediating the expression level of PEX2, and excessive hydrogen peroxide generated from peroxisomal β-oxidation suppresses liver ATGL activity and causes hepatic TAG accumulation (9). Therefore, upregulation of peroxisomal β-oxidation plays a pathogenic role in hepatic steatosis and oxidative injury. It is suggested that targeting peroxisomal β-oxidation might be a promising pathway in treating fatty liver and improving IR (6).

Peroxisomal β-oxidation can be induced by fasting, diabetes, HFD feeding, and hypolipidemic drugs and under the control of peroxisome proliferator activator receptor α (10, 11, 12, 13, 14), which plays an important role in the development of hepatic steatosis. However, it is not clear whether there exists a triggering mechanism for the induction of peroxisomal β-oxidation; addressing the potential triggering mechanism will help to understand the root cause of fatty liver and related metabolic diseases as caused by disorder in fatty acid metabolism. It was reported that hydrogen peroxide acted as a signal molecule inducing transcriptional activation of PEX genes and stimulating biogenesis of peroxisomes; however, it did not affect the enzymes in peroxisomal β-oxidation (30). Previous report suggests that upregulation in fatty acid ω-oxidation or treatment with LC-DCAs results in induction of peroxisomal β-oxidation, whereas suppression of fatty acid ω-oxidation significantly lowers peroxisomal fatty acid oxidation in isolated hepatocytes treated with CFB (20). The *in vitro* experiments provide strong evidence that LC-DCAs might play an important role in inducing peroxisomal fatty acid oxidation. However, whether endogenous LC-DCAs play a role in inducing peroxisomal β-oxidation *in vivo* is not verified. Our results indicate that administration of LC-DCAs rapidly and robustly induces peroxisomal β-oxidation and causes hepatic TAG accumulation in rodents through the metabolites acetyl-CoA and hydrogen peroxide, which is in agreement with the results with isolated hepatocytes and well supports the proposed role of LC-DCAs in regulating peroxisomal fatty acid oxidation.

LCDAs are the product of fatty acids subjected to ω-oxidation and were discovered in animals and humans in 1930s (31), whereas the pathophysiological roles of this kind of fatty acids are not clearly demonstrated. Early work by Björkhem (32) indicated that under normal condition, 10% fatty acids were subjected to ω-oxidation and might be remarkably induced under ketogenic conditions, who further proposed that the generation of LC-DCAs might facilitate oxidation of excessive FFAs. Another work by Kam *et al*. (33) suggested that the contribution of ω-oxidation to the initial oxidation of palmitate by liver slices was estimated to be between 8% and 11% and the oxidation of laurate between 17% and 21%. Later, LC-DCAs were reported to be able to suppress mitochondrial FAO through uncoupling oxidative phosphorylation (34). LC-DCAs were also considered to be gluconeogenic precursors that might be used for biosynthesis of glucose through generation of succinate (35, 36). It was reported that LC-DCAs showed antiketogenic effects and administration of LC-DCAs to fasting mice rapidly and robustly decreased plasma KB, indicating that endogenous LC-DCAs might play a negative role in regulating mitochondrial fatty acid oxidation and KB formation (37, 38). Our previous studies suggested that a physiological role of fatty acid ω-oxidation was to provide substrates for oxidation in peroxisomes for the purpose of generating succinate and regulating mitochondrial FAO through elevating the redox state of NADH in fasting as well as diabetic animals (23, 39). The study demonstrates a novel pathophysiological role of LC-DCAs, and the results suggest that administration of LC-DCAs in liver results in induction of peroxisomal β-oxidation, which negatively regulates mitochondrial FAO and increases hepatic TAG deposition.

It is generally accepted that LC-DCAs are exclusively metabolized by peroxisomal FAO system (3, 35), and short DCAs after metabolism can be used for glucose production or excreted in urine (35). Therefore, it is reasonable to assume that accumulation of LC-DCAs results in upregulation of peroxisomal β-oxidation, which might act as a feedback mechanism and negatively regulates intracellular level of LC-DCAs, and avoids uncontrolled high level of peroxisomal fatty acid metabolism. However, it should be noted that LC-DCAs must be activated to the CoA thioesters by a microsomal dicarboxylyl-CoA synthetase before entering into peroxisomes for degradation (40). It was reported that the activity of dicarboxylyl-CoA synthetase was not stimulated by CFB, a well-known drug in inducing peroxisomal β-oxidation (40), indicating that dicarboxylyl-CoA synthetase might be the rate-limiting enzyme for metabolism of LC-DCAs when intracellular level of this kind of fatty acids is very high. Enhancing the catalytic activity of dicarboxylyl-CoA synthetase in liver might be an effective pathway in removing the unphysiologically high level of LC-DCAs under ketogenic conditions, thereby lowering peroxisomal β-oxidation and improving hepatic steatosis.

This study reveals a novel crosstalk between fatty acid ω-oxidation and peroxisomal β-oxidation in animals. In mammalians, LC-DCAs are generated from mono fatty acids that are catalyzed by CYP4A1, followed by ADH and aldehyde dehydrogenase (15, 16). As fatty acid ω-oxidation is induced under the conditions of fasting, diabetes, and HFD feeding, which releases LC-DCAs and induces peroxisomal β-oxidation, we propose that induction of fatty acid ω-oxidation and elevation in endogenous LC-DCAs might play a pathological role in the development of fatty liver and IR through upregulation of peroxisomal β-oxidation under the condition of fasting, diabetes, or obesity. Besides, administration of fibrate drugs also induces fatty acid ω-oxidation and peroxisomal β-oxidation in animals and possibly in humans (19, 20), indicating that endogenously generated LC-DCAs might play a role in the development of hepatic steatosis and oxidative stress as caused by hypolipidemic drugs through induction of peroxisomal β-oxidation. It is intriguing to note that aspirin, a classical nonsteroidal anti-inflammatory drug, induces both peroxisomal β-oxidation and fatty acid ω-oxidation (41, 42, 43), and aspirin might cause oxidative liver injury though peroxisome-generated reactive oxygen species (ROS). Our preliminary results suggest that liver LC-DCAs increase significantly and dose-dependently in the mice treated with aspirin, which remarkably induces peroxisomal β-oxidation and increases liver ROS levels, indicating that fatty acid ω-oxidation and peroxisomal β-oxidation might be involved in aspirin-induced hepatic oxidative injury. Further studies will be carried out to demonstrate the potential role of fatty acid ω-oxidation–peroxisomal β-oxidation–ROS axis in aspirin-induced oxidative liver injury.

Clinical data on the levels of LC-DCAs in human liver are lacking; however, solid evidences suggest that urinary LC-DCAs increase significantly in diabetic and fasting individuals (44, 45, 46), indicating accelerated ω-oxidation of endogenous fatty acids in human liver and kidney under ketogenic conditions. More clinical data are required to determine the content of LC-DCAs in human liver, especially in diabetic and obese subjects, which will help to verify whether endogenously generated LC-DCAs might play a role in inducing peroxisomal β-oxidation and hepatic steatosis as they work in mice. In this study, administration of 4-MP, a specific inhibitor for ADH, significantly reduced LC-DCA level in the liver of the fasting mice and lowered peroxisomal β-oxidation and hepatic TAG level, which well supports the proposed mechanism. Therefore, specific inhibition of the key enzymes involved in fatty acid ω-oxidation (CYP4A1, long-chain ADH) might be a potential pathway in treating fatty liver and improving IR through regulating hepatic level of LC-DCAs and peroxisomal β-oxidation.

Endogenously generated LC-DCAs might also be involved in alcohol-induced fatty liver and oxidative injury through induction of peroxisomal fatty acid oxidation. It was reported that intake of alcohol stimulated liver fatty acid ω-oxidation and LC-DCAs increased significantly in the liver of animals (47, 48). In the meantime, peroxisomal β-oxidation was also unregulated in the livers of the animals and humans receiving alcohol (48), which suggested that endogenous LC-DCAs might play a role in alcohol-induced hepatic lipid accumulation and oxidative stress. Administration of 4-MP or pyrazole, well-known inhibitors for liver ADH, significantly reduced hepatic lipid levels in animals receiving alcohol (49, 50), which well supported the proposed mechanism. Therefore, it is suggested that excessive generation of LC-DCAs might play a role in alcohol-induced hepatic TAG accumulation through induction of peroxisomal β-oxidation, and small molecules that specifically target the key enzymes in fatty acid ω-oxidation will be promising agents in treating alcoholic fatty liver by reducing endogenous LC-DCAs and suppressing peroxisomal β-oxidation, which increases fatty acid burning in mitochondria and attenuates hepatic accumulation of TAG.

## Experimental procedures

### Materials

Coenzyme A sodium salt, acetyl-CoA, Percoll were purchased from Sigma. CFB, 4-MP, DCA_12_, and C12 were from Tokyo Chemical Industry. All other chemical reagents used were of analytical grade or better.

### Animal studies

C57BL/6J mice at the age of 8 weeks were purchased from Slac Laboratory Animal Co Ltd. Standard rodent diet (12% fat by calories) containing 5% (w/w) C12 or 5% (w/w) DCA_12_ was supplied by Slac Laboratory Animal Co Ltd. All the animals were housed in single cage with free access to food and water under controlled temperature (22 °C) and light (12 h of light and 12 h of dark). Normal group (N) was fed standard rodent diet (12% fat by calories), C12 group was fed standard rodent diet containing 5% (w/w) C12, whereas the mice in DCA_12_ group received rodent diet containing 5% (w/w) DCA_12_. All the mice were fed the corresponding diets for 7 days. For the purpose of studying the role of endogenous LC-DCAs in inducing peroxisomal β-oxidation under ketogenic conditions, the mice were fasted for 48 h. To induce fatty acid ω-oxidation, CFB at a dose of 240 mg/kg was administered to the mice by gavage at the beginning of fasting, the mice were treated for 2 days, twice per day at 8 AM and 6 PM, respectively. To suppress ADH, 4-MP at a dose of 200 mg/kg was administered to the fasting mice by intraperitoneal injection, once per day at 10 AM. After the experiments, all the mice were bled from the eyes and then sacrificed under anesthesia. Livers were removed quickly and stored in liquid nitrogen immediately. Histological analysis was performed according to the protocol as described previously (4). All the animal studies were approved by the Animal Care Committee of Hunan University of Science and Technology.

### Isolation of peroxisomes

Peroxisomes were isolated by the method of differential centrifugation and further purified by a Percoll gradient according to the methods as described previously (51, 52).

### Measurement of LC-DCAs in liver tissue

Liver LC-DCAs were extracted by the method as described by Mortensen (53), and the level of LC-DCAs was measured by GC–MS and expressed as the sum of DCAs with chain length of C10–C22 (44, 53).

### Quantitative real-time PCR

Total RNA was extracted from liver tissues with TRIzol reagent (Life Technologies Corporation). RNA was reverse-transcribed with standard reagents (High-capacity Reverse Transcription Kits; Applied Biosystems) using random primers. Complementary DNA was amplified in a 7500 Fast Real-time PCR System using 2× SYBR Green Supermix (Applied Biosystems). mRNA expression levels normalized to 18S rRNA were expressed using the comparative delta CT method. The primers used in this study are shown in Table S1.

### Biochemical analysis

Liver FFA concentration was determined using a colorimetric kit based on ACS-ACOX method (Wako). Plasma TAG was determined by commercial kit according to the manufacturer’s instructions (Wako). Plasma KB was determined enzymatically and expressed as the sum of βOHB and AcAc (54). Liver acetyl-CoA was determined enzymatically according to the method as described previously (55). Liver long-chain acyl-CoAs were extracted and determined by the method of Tubbs and Garland (54). Liver malonyl-CoA was analyzed by HPLC as described previously (56). Liver ADH activity was assayed by the method as described previously (57). Peroxisomal β-oxidation was assayed by acyl-CoA-dependent NAD^+^ reduction in the presence of KCN as reported by Lazarow (58). ACOX1 activity was determined according to the method as described previously (4). Liver acetyl-CoA thioesterase activity was determined by the method of Hunt (59). Liver ATGL activity was assayed by the method as described previously (9). Liver TAGs were extracted by the method of Bligh and Dyer (60) and determined with a commercial kit (Wako). Liver catalase, hydrogen peroxide, thiobarbituric acid–reactive substance citrate, and acetate were determined by commercial kits (Sigma). Protein concentration was measured by Bio-Rad DC protein assay kit.

### Statistical analysis

Data are presented as mean ± SEM. n = 6 to 8 for all the groups. The significance of differences was evaluated using Student's test by SPSS, version 18.0 (IBM). *p* < 0.05 was considered statistically significant.

## Data availability

All data are contained within the article.

## Supporting information

This article contains supporting information.

## Conflict of interest

The authors declare that they have no conflicts of interest with the contents of this article.
