# Supplementary material for: Long-chain dicarboxylic acids play a critical role in inducing peroxisomal β-oxidation and hepatic triacylglycerol accumulation
Source: J Biol Chem. 2023 Aug 19;299(9):105174. doi: 10.1016/j.jbc.2023.105174 (PMC10494467; doi:10.1016/j.jbc.2023.105174)
Supplement: Table S1 [file mmc1.docx]

**Table S1. Primers used for RT-PCR**

| **Primer** | **sequences (5’-3’)** |
| --- | --- |
| ACSLC1-F  ACSLC1-R | GGAAGCCAAACCAGCCCTAT  ATCCTTGCTCGACGATCACC |
| ABCD1-F  ABCD-R | ATGAAGGAAGAGGAGCTGGT  TGGAACATCTCGTACACCCT |
| ACOX1-F  ACOX-R | ATTCAAGACAGAGCCGTGCA  TCAAAGGCATCCACCAAAGC |
| L-BP-F  L-BP-R | GGGTGGCTGCTGAATCTCTT  CCGCCACCAAACTGTAGTCA |
| Thiolase-F  Thiolase-R | AGGCTTCAAGAACACCACCC  CTCAGAAATTGGGCGATGCG |
| ACOT12-F  ACOT12-R | ATAGACCAGGTGAGCGAGGA  TGCCAGCAAAGTAAGGGAGG |
| PEX2-F  PEX2-R | TGCCTCTAGTAGTTCCACGT  TAAACTGGGACCACACAAGC |
| CYP4A1-F  CYP4A1-R | AACCGTGCTTGTCAACTTGC  AGCTGTCCCCATTCTCCATT |
| LCADH-F  LCADH-R | GTGTGTGTGCCCTACTGACA  ACAGAGGTTTGTGAGAGGGC |
| LCALDH-F  LCALDH-R | ACGTGGTGGTGATGAAGGTG  GAACGCCACTTTGTCCACAC |
